# Photobiomodulation of human osteoblast‐like cells *in vitro* by low‐intensity‐pulsed LED light

**DOI:** 10.1002/2211-5463.12877

**Published:** 2020-05-28

**Authors:** Nahum Rosenberg, Raya Gendelman, Nesreen Noofi

**Affiliations:** ^1^ Laboratory of Musculoskeletal Research Rambam Health Care Campus and Ruth & Bruce Rappaport Faculty of Medicine Technion – Israel Institute of Technology Haifa Israel

**Keywords:** cell death, cell maturation, LED, osteoblast‐like cells, pulse light photobiomodulation

## Abstract

Visible light irradiation is an emerging area in regenerative medicine research. We hypothesized that low‐intensity‐pulsed LED light irradiance may exert photobiomodulatory effects on cultured osteoblast‐like cells. To test this hypothesis, we investigated cell proliferation and markers of cell maturation and metabolic activity following pulsed LED irradiance. Monolayer explant cultures of human osteoblast‐like cells were exposed four times in 24‐h intervals to 2 min of pulsed white LED irradiance of 2.4–2.5 mW·cm^−2^ and its different spectra of 0.2–0.5 mW·cm^−2^ (frequency range of 10–40 Hz). Cell proliferation was estimated from microscopic cell counting and cell death by *lactate dehydrogenase* activity in culture media (measured by a colorimetric method). The early markers of osteoblast maturation and metabolic activity, that is, cellular *alkaline phosphatase* activity and *osteocalcin* content, were measured using a colorimetric method and ELISA, respectively. Irradiance of 40 Hz caused the highest increase in cell number (*P* < 0.01). *Osteocalcin* content in cells decreased following 40 Hz and 10 Hz irradiance (*P* < 0.05). The 40 Hz blue range irradiance (diffuse transmittance 420–580 nm, maximal cell irradiance 0.5 mW·cm^−2^) caused a decrease in *alkaline phosphatase* cellular activity (*P* < 0.001) and an increase in media *osteocalcin* content (*P* < 0.05). The 40 Hz green range (diffuse transmittance 560–650 nm, maximal cell irradiance 0.4 mW·cm^−2^) irradiance caused an increase in the number of cells and in cell death. In summary, pulsed (40 Hz) white light irradiance has photomodulatory effects, with its green range spectrum affecting cell proliferation and cell death, and its blue range spectrum affecting cellular maturation and metabolism. The results indicate a low‐intensity threshold of photobiomodulation of osteoblast‐like cells *in vitro*.

## Graphical abstract

Low‐intensity (0.4–0.5 mW·cm^−2^) 20–40 Hz‐pulsed LED irradiance effect on proliferation and maturation of human osteoblast‐like cells was investigated. We found that 40 Hz‐pulsed irradiance is optimal for photomodulation of these cells, when the blue spectrum (420–580 nm) causes a decrease in early maturation and the green spectrum (diffuse transmittance 560–650 nm) increases cell proliferation.

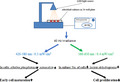

**Abbreviations**
AFSC: amniotic fluid stem cells
ALP: alkaline phosphatase
ANOVA: analysis of variance
ATP: adenosine triphosphate
BSP: bone sialoprotein
ChR2: channelrhodopsin‐2
cm: centimeter
DMEM: Dulbecco's modified Eagle Medium
ELISA: enzyme‐linked immunosorbent assay
FGIN‐1‐27: N,N‐Dihexyl‐2‐(4‐fluorophenyl)indole‐3‐acetamide)
He‐Ne: helium–neon
HEPES: 4‐(2‐hydroxyethyl)‐1‐piperazineethanesulfonic acid
Hz: hertz
J: joule
kDa: kilodalton
LDH: lactate dehydrogenase
LED: light‐emitting diode
MAPK: mitogen‐activated protein kinase
MSC: mesenchymal stem cells
Mw: milliwatt
nm: nanometer
NO: nitric oxide
PDGF: platelet‐derived growth factor
TSPO: translocator protein (18 kDa)
U: unit
v:v: volume per volume

## Introduction

Visible light irradiation is an emerging area in regenerative medicine research. The focus of the numerous reports in this field is to develop a technique for cellular activation by controlling the light source, light wavelength, and irradiance in order to illicit proliferation, differentiation, and different aspects of metabolic activities of cells *in vitro*.

It was shown that red light He‐Ne laser (632 nm wavelength) with 5.3 mW power that was applied directly to injured skeletal muscles of toads and rats affected the pathways of cell survival, enhanced angiogenesis, delayed apoptosis, and increased ATP production [1]. In another study, visible light with a bandwidth of 400–800 nm at a power of 4.8 J·cm^−2^ caused a significant increase (1.78‐fold) in the colony growth of cultured mesenchymal stem cells (MSCs). It was concluded that proliferation is a direct effect of absorption of various wavelengths by cellular chromospheres [2]. Other studies investigated how a light beam can affect cell regeneration and showed positive results (*in vitro*) in neurons and fibroblasts [3, 4].

Several theories exist regarding the mechanism of cellular photobiomodulation. In neurons, it was hypothesized that the expression of rhodopsin channels (ChR2) caused an induction of rapid depolarized current when exposed to blue light (450–490 nm). The light was shown to mediate the generation of synaptic events [3]. Neurons were also observed to express halorhodopsins Cl^−^ (light‐gated ion pump channels), which, when exposed to red light (580 nm), caused a hyperpolarization reaction and inhibition of firing of action potential [5]. In keratinocytes, it was found that blue light (up to 453 nm) reduced cellular proliferation and induced differentiation, while light wavelengths of 630–940 nm had no distinct effect. It was also observed that cellular ATP increased significantly with blue light (453 nm) by releasing NO from nitrosated proteins in the mitochondria [5]. Additionally, evidence exists that blue light irradiation causes an increase in cellular metabolism via succinate dehydrogenase and complex 2 of the electron transport chain [6]. It was also shown that low‐intensity red light (628 nm) irradiance stimulated proliferation in fibroblasts. It was suggested that red light caused an increase in MAPK11, which upregulated the P38 MAPK pathway and furthermore upregulated PDGF and other growth signaling pathways, leading to fibroblast proliferation, collagen synthesis, and enhanced wound healing [4]. Evidence exists showing that red light influences the respiratory chain by cytochrome C oxidase, which acts as a photoreceptor and affects mitochondrial metabolism [7]. But other reports show that fibroblasts respond (by increased proliferation) especially to a different range of green photobiomodulation by LED irradiance of 570 nm. [8, 9] This inconsistency of observations from different studies could be attributed to the different experimental setups [10].

Although it has been shown that visible light is effective in cellular activation in general, little has been studied regarding the effect of light on cells in MSCs on the osteoblast pathway. One study investigated amniotic fluid stem cells (AFSCs) and their osteogenic differentiation following exposure to visible light from an LED array of blue (470 nm), green (525 nm), yellow (600 nm), and red (630 nm) light ranges. Overall, light irradiation enhanced osteogenesis. Green light (525 nm) proved to enhance gene expression, and blue light (470 nm) enhanced the most characteristic osteoblast markers (calcium deposition and ALP activity) compared to other bandwidths of visible light photobiomodulation [11]. It is apparent from different studies that blue light (453 nm) is effective in enhancing the differentiation of mesenchymal stem cells into osteoblasts through flavin–protein activation [6], while the osteogenic effect of red light (630 nm) involves cytochrome C oxidase [7].

To induce the maximum cellular effect of light, it has been proposed that irradiation should be conducted in a pulsed protocol. Several studies have chosen pulsed over continuous light stimulation as a method of evoking cell signaling. It was concluded that ChR2 channels, when activated with a sequence of brief pulses, consistently elicited precise and reproducible synaptic events in neurons over a period of time [3]. Furthermore, another study showed that ChR2 channels, which act distinctively as outward rectifying channels, conducted passive currents when irradiated with 2 Hz‐pulsed blue light generated inward as photocurrents [12]. Similarly, an increase in electron transfer was noted when pulsed laser light evoked electrogenic events and redox changes in the cytochrome c oxidase in the mitochondria [7]. As an overall conclusion, it might be suggested that pulsed light irradiance on cells elicits variable cellular responses, for example, enhanced cell proliferation and/or differentiation.

Therefore, the objective of this study was to investigate the effects of pulsed white LED light on human osteoblast‐like cells *in vitro*. Previous evidence exists that these cells are metabolically sensitive to mechanical [13] and electromagnetic [14] stimulation applied at frequencies of 20–60 Hz, probably through transmembrane electric currents. Therefore, we hypothesize that LED light exposure that might also be mediated by transmembrane electric currents at the same frequency range of stimulation should induce similar effects on cellular synthetic activity, maturation, and death rate. We also hypothesized that various ranges of pulsed LED light spectrum induce different basic cellular activities, that is, cell maturation, synthetic activity, and cell death rate.

## Materials and methods

**Fig. 1:**
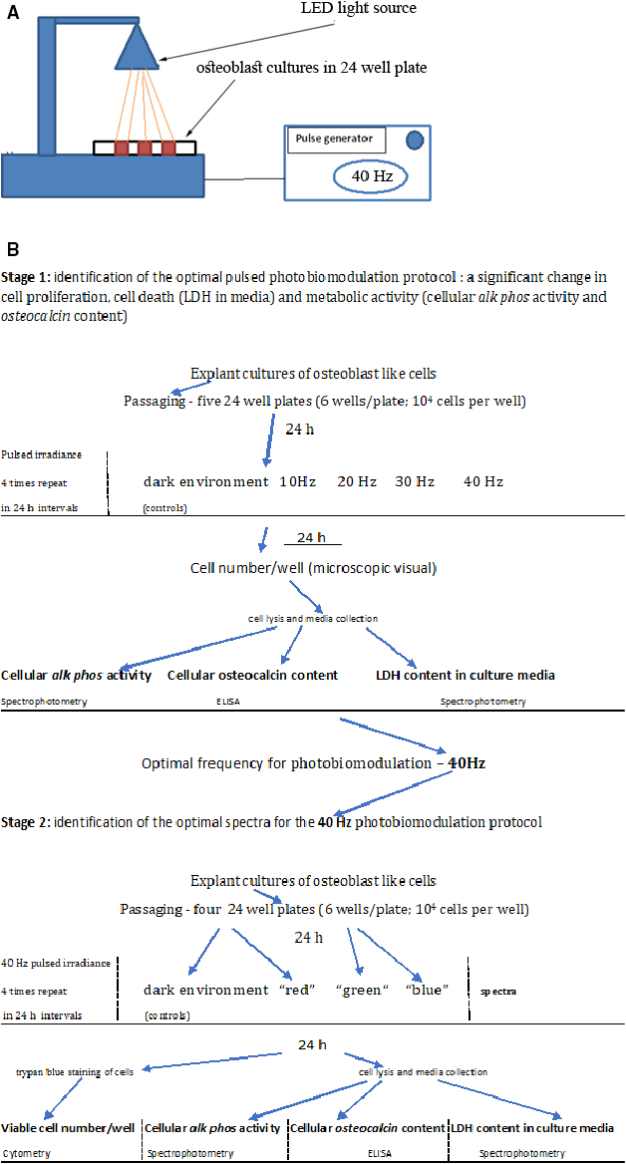
Schematic representation (A) and flowchart (B) of the experimental setup and procedure.

According to previous research protocols of photobiomodulation in osteoblasts, we investigated the cellular response by measuring cell proliferation, cell death, cell viability, cell maturation (by alkaline phosphatase activity), and osteoblast metabolic activity (by cellular osteocalcin content) [10]. The experiments were performed in two stages. In Stage 1, the optimal frequency of pulsed white LED photobiomodulation on the stimulation or inhibition of different cellular activities was determined, and in Stage 2, an attempt was made to determine the effect of different parts of the white LED light spectrum on different cellular activities at the optimal photomodulation frequency found in Stage 1 of this experiment (Fig. 1).

### Stage 1

We used primary cultures of human osteoblast‐like cells. Briefly, chips of cancellous bone were prepared from disposable bone samples that were collected from the proximal femora of six donors (two men, four women, age range 65–75 years) during fractured hip arthroplasties. No osteoarthritic changes in the femoral head were evident under direct inspection (no gross cartilage damage was seen on the articular surface), and the samples were collected from the femoral canal—at least 4 centimeters distant from the subchondral area. The bone samples, each 2–3 grams in total, were incubated in osteogenic medium [15, 16]: DMEM with heat‐inactivated fetal calf serum (10%), 20 mm HEPES buffer, 2 mm l‐glutamine, 100 μm ascorbate‐2‐phosphate, 10 nm dexamethasone, 50 U·mL^−1^ penicillin, 150 μg·mL^−1^ streptomycin, at 37 °C in humidified air with 5% CO_2_ (v:v) for 20–30 days. As previously described, human osteoblast‐like cells grew from the chips as a primary cell culture adherent to the plastic tissue culture plates (non‐pyrogenic polystyrene) [12]. The study methodologies conformed to the standards set by the Declaration of Helsinki. The use of these cells for the experiments was approved by the Institutional Ethics Committee (Rambam Health Care Campus No. A1240). Each donor provided written informed consent for the use of their tissues. The collection site of bone samples was distant from the subchondral bone area to avoid local metaplastic effects.

The human bone cell cultures obtained by this standard method were previously shown to express osteoblast‐like characteristics, that is, polygonal multipolar morphology, expression of the enzyme alkaline phosphatase, synthesis of a collagen‐rich extracellular matrix with predominantly type I collagen, and also small amounts of collagen types III and V, as well as non‐collagenous proteins such as sialoprotein (BSP) and osteocalcin [15, 16]. Additionally, these cells demonstrate matrix mineralization *in vitro* and bone formation *in vivo*. Furthermore, we had also previously shown osteoblast characteristics of these cells, including positive Von Kossa staining, synthesis of osteopontin, characteristic multipolar morphology, adherence to plastic surface, osteocalcin content, and cellular alkaline phosphatase activity [17]. In the present study, cells were allowed to migrate from the bone chips into the medium and proliferate in 75‐cm^2^ culture flasks for 21 days (explant cultures). The cells were then placed into 24‐well plates, where each well was seeded with 10^4^ cells. The cultured samples were exposed to the pulsed LED light protocol described below. The cultures were maintained in the dark between exposure to the light protocol to prevent uncontrolled light interactions. Six samples (six wells in 24 different well plates) were exposed to different experimental conditions.

### LED light exposure setup

Well plates with cultured osteoblast‐like cells were exposed to a horizontally directed LED light source in a dark environment from a distance of 6 cm (Fig. 1). This distance was chosen to create irradiance on the cells in the range of 2.4–2.5 mW·cm^−2^. The membranal stimulation can be elicited by white light intensity as low as 0.6–3.2 mW·cm^−2^ in the light‐sensitive neural cells [18]. We chose irradiance intensity empirically at a higher level of magnitude range of around 2.0–3.0 mW·cm^−2^ because the osteoblast is probably less sensitive to light than visual neurons. The irradiance was applied to different culture samples at 10, 20, 30, and 40 Hz. Control cultures were kept in dark conditions.

### LED light exposure protocol

The cells were placed into 24‐well plates. Each well was seeded with 10^4^ cells. Six replicate wells were prepared for each experimental condition. Following placement, the cells were incubated under the same basic media conditions in these media for 24 h and subsequently exposed four times to 2 min of light irradiation at 24‐h intervals. This protocol is empirical and similar to the previous protocol of biomechanical osteoblast stimulation by vibration in the 20–60 Hz range of frequencies [19].

### Biochemical assays

The cells in each culture sample were counted microscopically. The average number of cells in three low‐power microscopic fields was considered as representative for each experimental condition. Following cell counting in each culture sample, the media were collected for further assays and the adherent cells were washed by PBS and lysed in 20% Triton X‐100 by three cycles of freezing to −20 °C and thawing at 20 °C.

Alkaline phosphatase (ALP) activity (a marker for young osteoblast maturation) was determined in lysed cells following incubation with P‐nitrophenyl phosphate substrate at a concentration of 20.5 mmol·L^−1^ in the presence of transphosphorylating buffer 2‐amino‐2‐methyl‐1‐propanol at (pH = 10.35) by 410 nm wavelength spectrophotometry [20]. The range of the assay was 11–1000 U·L^−1^. The results were normalized to cell numbers in each sample as microscopically counted (described above). We used the exposure to ALP inhibitor (activin 100 ng·mL^−1^) on osteoblast cultures as a positive control. We had shown previously that activin (100 ng·mL^−1^) causes a significant decrease in osteoblast ALP activity [18].

*Osteocalcin* cellular content (a marker for synthetic osteoblastic activity) in cells was assayed by ELISA (N‐MID^®^ Osteocalcin ELISA, Immunodiagnostic Systems, East Boldon, UK) in a Cobas e analyzer (Roche Diagnostics Mannheim Germany). Briefly, in the lysed cell samples, ‘sandwich’ complexes were formed following incubation with a 20 μL biotinylated monoclonal N‐MID osteocalcin‐specific antibody and a monoclonal N‐MID osteocalcin‐specific antibody labeled with a ruthenium complex. Then, second incubation with streptavidin‐coated microparticles was done. The reaction mixture was aspirated into measuring cells where microparticles were captured magnetically to an electrode. Unbound substances were removed. Voltage was applied to the electrode to induce a chemiluminescent emission measured by a photomultiplier (according to the N‐MID® kit instructions). Results were determined via a calibration curve generated by 2‐point calibration and a master curve [21]. The range of the assay was 0.5–3.0 ng·mL^−1^. The results were normalized to cell number in each sample as counted microscopically (described above).

*Lactate dehydrogenase* (LDH) activity (a marker of cell death) in the collected culture media was determined by 340 nm wavelength spectrophotometry of the reduced NAD, that is, measurement of the oxidation of L‐lactate to pyruvate at pH = 8.55 in a Tris buffer (15.3 µmol·L^−1^). This value is directly proportional to LDH activity [22, 23]. The range of the assay was 0–600 U·L^−1^. The LDH content in the culture media before the experiment was 10.80 U·L^−1^. This value was deduced from the experimental results presented. For a positive control, we used cells kept in the dark and treated with FGIN‐1‐27(10^−5^ m), which is a synthetic TSPO (18 kDa mitochondrial translocator protein) ligand. We had shown previously that the FGIN‐1‐27(10^−5^ m) causes a significant increase in culture media LDH content [24].

In both ALP activity and LDH activity, a spectrophotometer (Dimension AR IMT 110V/60 Hz, Dade Behring, Inc. Newark, DE) was used.

The results of Stage 1 indicated that the main cellular effects, that is, effect on cell numbers and cellular metabolic activity as expressed by osteocalcin content, are exhorted by pulsed white LED irradiation at 40 Hz. Therefore, in Stage 2 of the experiments, we investigated the contribution of different parts of the spectrum on cells following exposure to 40 Hz‐pulsed LED irradiation.

### Stage 2

Cultured samples from the same origin as in the Stage 1 experiment were used.

**Fig. 2:**
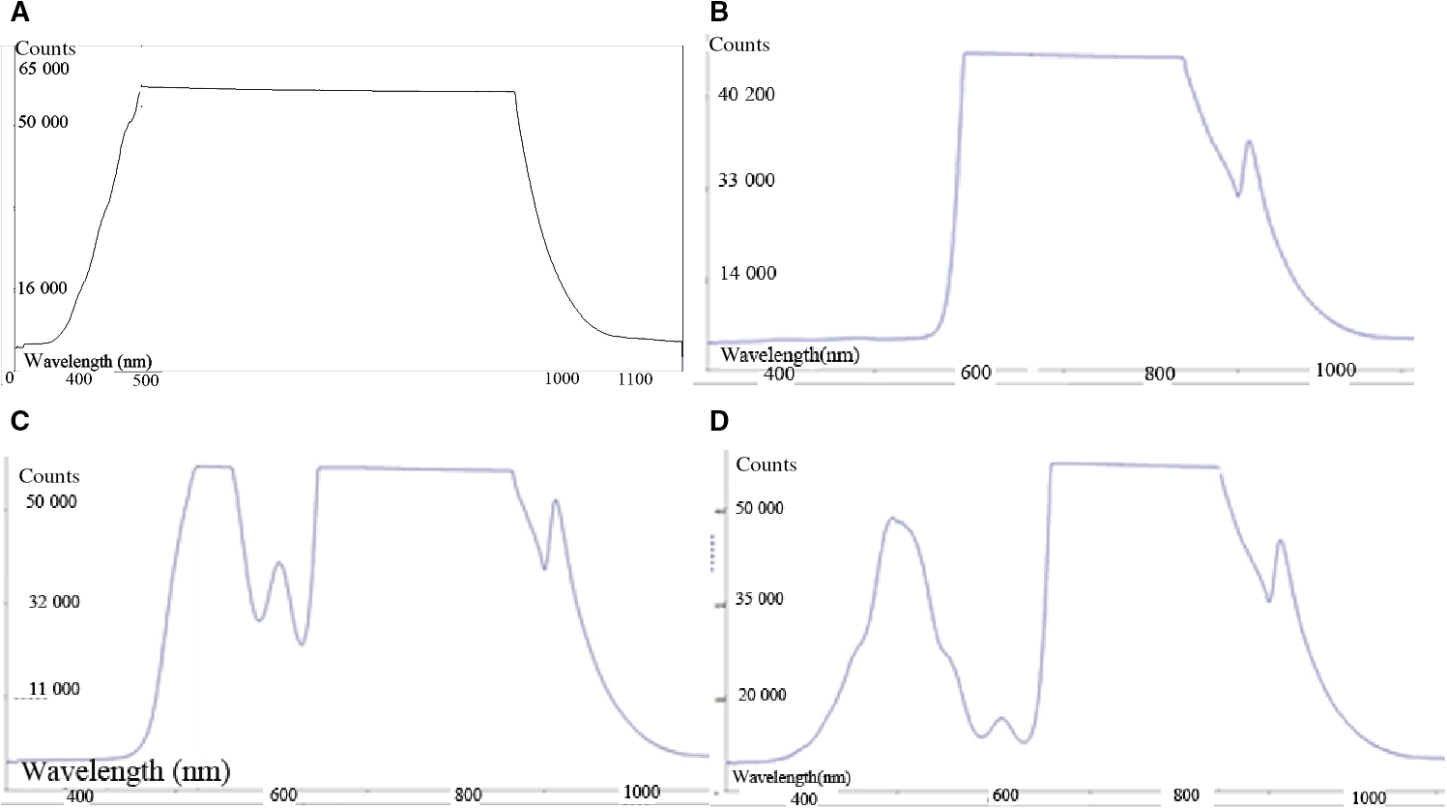
Spectra of LED 40 Hz‐pulsed light for irradiance of cultured cell samples. (A) unfiltered light source, (B) red filtered—593–840 nm, (C) green filtered—560–650 nm, (D) blue filtered—420–580 nm.

We used the same photobiomodulation of cells setup as in Stage 1 with white LED light pulses of 40 Hz. The light was applied through red (diffuse transmittance 593–840 nm, maximal cell irradiance 0.2 mW·cm^−2^), green (diffuse transmittance 560–650 nm, maximal cell irradiance 0.4 mW·cm^−2^), and blue (diffuse transmittance 420–580 nm, maximal cell irradiance 0.5 mW·cm^−2^) filters (Fig. 2); control cultures were kept in dark conditions. The irradiance intensity originating from the same LED source as in Stage 1 varied in the same range following light filtration according to the physical properties of the light filters used, representing only the filtered light irradiance.

To make a quantitative assessment of the viable cells in each culture, the cells were counted cytometrically, and the number of viable cells was measured by the dye exclusion method using trypan blue staining and counted cytometrically using the TC20^TM^ Automated Cell Counter (Bio‐Rad Laboratories Ltd.). The measurements were made on the suspension of cells following their removal from the well surface.

LDH activity in the culture media, cellular osteocalcin content, and ALP activity were measured using the same methods as described in Stage 1 of this experiment.

To simplify the description of the experiments, we summarize the steps of both stages in Fig. 1B.

### Statistical analysis

All data were of the quantitative type. The independent variables were the frequencies of the light exposure protocol in Stage 1 of the study and the wavelengths of the light exposure at 40 Hz of light irradiance in Stage 2 of the study. When normal distribution of numeric results was found by the Kolmogorov–Smirnov test, the one‐way ANOVA test was used followed by an appropriate post hoc comparison (for a comparison of pairs of result groups); otherwise, a non‐parametric test for comparison was utilized. A *P* value less than 0.05 was considered as statistically significant. [25] The calculations of the statistical comparisons were done using sigmastat software (version 2, SPSS Inc., Chicago, Il, USA).

## Results

### Stage 1

#### Cell numbers per microscopic field

**Fig. 3:**
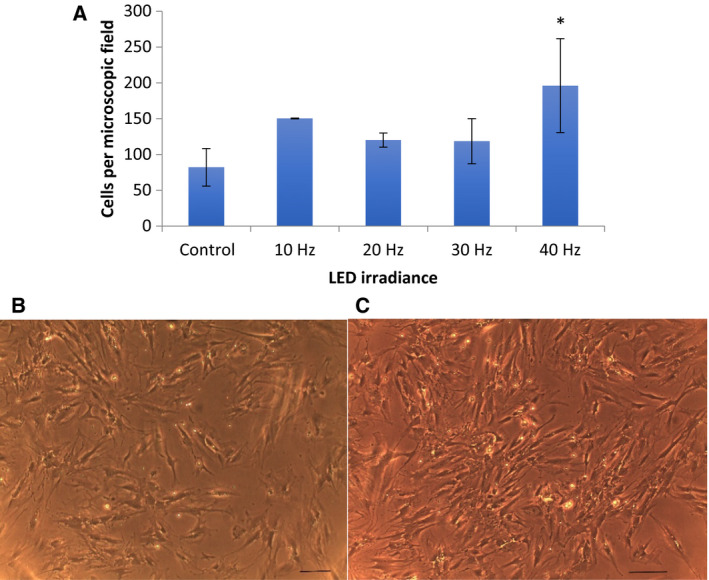
Cell number per microscopic field. (A) Mean values with standard deviation (vertical bars) are presented. Significantly higher number of cells in cultures observed (images B, C) following pulsed white LED irradiance at 40 Hz frequency (*P < 0.01, *n* = 6). (B) Representative microscopic image of a control culture (scale 20 µm). (C) Representative microscopic image of a cell culture following exposure to 40 Hz LED irradiance protocol (scale 20 µm).

We found the highest number of cells in samples following exposure to 40 Hz‐pulsed irradiance (mean 196.00 ± 65.48 SD cells per microscopic field vs. 82.000 ± 26.153 SD in controls, *P* < 0.01). The 10 Hz, 20 Hz, and 30 Hz groups also showed a significant increase in cell numbers per culture sample in comparison with the control (mean 150.333 ± 0.577 SD, 120.000 ± 10.000 SD, and 118.333 ± 31.501 SD cells per microscopic field, respectively, *P* < 0.01, ANOVA; Fig. 3).

#### Alkaline phosphatase activity in cells

No significant difference in cellular ALP activity between the groups was found (the median values for each group were 0.239 ± 0.071 SD, 0.213 ± 0.001 SD, 0.248 ± 0.001 SD, and 0.210 ± 0.038 SD U/L/cell/microscopic field for the 10 Hz, 20 Hz, 30 Hz, and 40 Hz groups, respectively, compared to 0.187 ± 0.111 SD U/L/cell/microscopic field for the controls, *n* = 6, *P* > 0.05, Kruskal‐Wallis one‐way analysis on rank test.

#### Osteocalcin content in cells

**Fig. 4:**
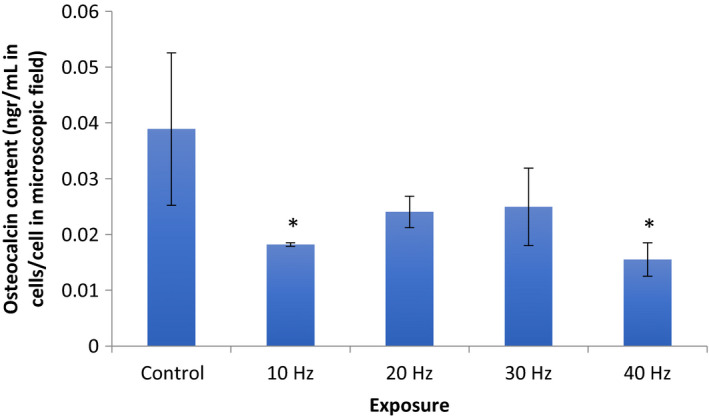
Cellular osteocalcin content in cells following different frequency regimes of irradiance. There is a significant decrease following the 10 Hz and 40 Hz irradiance in comparison with other experimental conditions and controls (*P < 0.05, *n* = 6). Mean values with standard deviation (vertical bars) are presented.

The samples exposed to 40 Hz and 10 Hz irradiance had significantly lower mean osteocalcin contents, with 0.015 ± 0.002 SD ng·mL^−1^ per cell and 0.0182 ± 0.01 SD ng·mL^−1^ per cell, respectively, in comparison with the controls with 0.0389 ± 0.0137 SD ng·mL^−1^ per cell (*P* < 0.05, ANOVA, *n* = 6). Twenty Hz and 30 Hz irradiance on samples showed similar mean values of 0.0240 ± 0.00281 ng·mL^−1^ per cell and 0.0250 ± 0.007 SD ng·mL^−1^ per cell, respectively (*P* > 0.05, ANOVA, *n* = 6; Fig. 4).

#### LDH activity in the media

LDH activity in the culture media before the experiment was 10.80 U L^−1^. This value was deduced from the experimental results presented.

There was no significant difference (*P* > 0.05, ANOVA, *n* = 6) between LDH activity in media among the cultured samples (mean 20.67 ± 1.53 SD, 20.33 ± 3.2 SD, 23.00 ± 3.00 SD, and 21.33 ± 0.11 SD U L^−1^ for the 10 Hz, 20 Hz, 30 Hz, and 40 Hz groups, respectively, compared to 20.67 ± 5.03 SD U L^−1^ for the controls).

### Stage 2

**Fig. 5:**
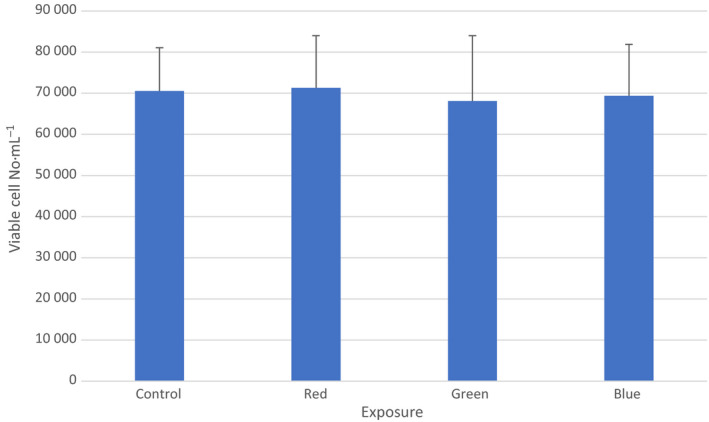
Cytometric count of cells. Mean values with standard deviation (vertical bars) are presented. No significant difference in the number of viable cells in all cultured samples was found (*P* > 0.05, *n* = 6). Filtered 40 Hz‐pulsed light predominant irradiance: red—593–840 nm, green—560–650 nm, blue—420–580 nm, control—cells kept in dark, positive control—cells kept in dark.

Cytometric measurements showed that there was no significant difference in the number of viable cells in all cultured samples (mean 70 540 ± 10 545 SD viable cells mL^−1^, 71 320 ± 112 680 SD viable cells mL^−1^, 68 100 ± 15 900 SD viable cells mL^−1^, and 69 356 ± 12 522 SD viable cells·mL^−1^ in the control exposed to the red range, green range, and blue range irradiance, respectively (*P* > 0.05, ANPVA, *n* = 6; Fig. 5).

Since no significant difference in the number of cells in all of the cultured samples was found, no normalization per number of cells in the culture in other assays was necessary.

#### Alkaline phosphatase activity in cells

**Fig. 6:**
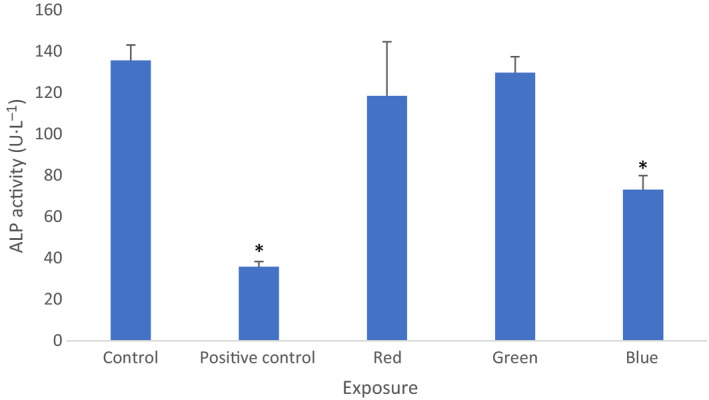
Cellular *alkaline phosphatase* (ALP) activity. There is a significant decrease following the blue light irradiance of cells in comparison with other experimental conditions (*P < 0.001). Mean values with standard deviation (vertical bars) are presented. Filtered 40 Hz‐pulsed light predominant irradiance: red—593–840 nm, green—56–650 nm, blue—420–580 nm, control—cells kept in dark, positive control—cells kept in dark and treated with activin 100 ng mL^−1^.

We found that samples exposed to the blue range light showed the lowest ALP activity level of mean 73.25 ± 6.652 SD U L^−1^ (*P* < 0.001, ANOVA, *n* = 6). The control, green light, and red light groups had mean activity values of 135.75 ± 7.411 SD, 118.5 ± 26.236 SD, and 129.75 ± 7.676 SD U L^−1^, respectively. Cells exposed to the positive control agent (ALP inhibitor, activin 100 ng mL^−1^) following the blue range light irradiance showed significantly lower ALP activity in comparison with the controls (mean activity 35.85 ± 2.53 SD U L^−1^, 73.25 ± 6.652 SD U L^−1^, respectively, *P* < 0.001, ANOVA, *n* = 6). This result suggests that cell irradiance with the blue light moderately reduced the cell maturation rate (Fig. 6).

#### Osteocalcin content in cells

**Fig. 7:**
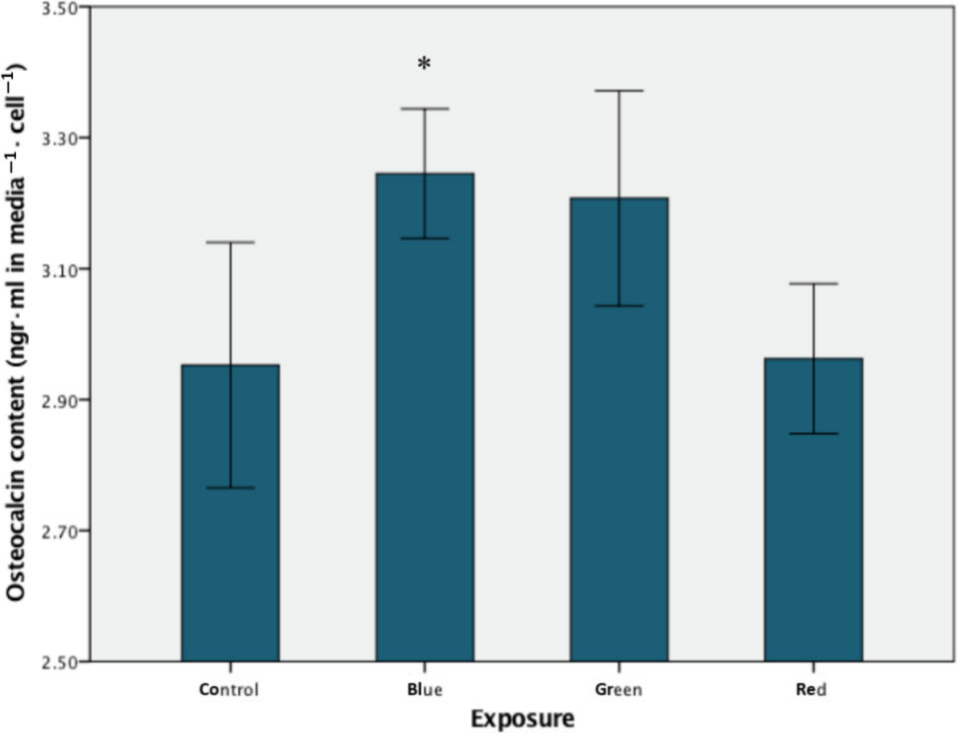
Cellular osteocalcin content. There is a significant increase following the blue range light irradiance of cells in comparison with the controls (*P < 0.05, *n* = 6). Mean values with standard deviation (vertical bars) are presented. Filtered light predominant irradiance: red—593–840 nm, green—560–650 nm, blue—420–580 nm.

Samples that were exposed to the pulsed 40 Hz blue range LED light showed a significantly higher level of cellular osteocalcin content in comparison with the controls (mean 3.245 ± 0.098 ng mL^−1^ compared to mean 2.953 ± 0.187 ng mL^−1^, *P* < 0.05, ANOVA, *n* = 6; Fig. 7). The red range light and green range light exposures had no significant effect on cellular osteocalcin content (mean 2.962 ± 0.114 SD ng mL^−1^ and mean 3.208 ± 0.164 SD ng mL^−1^, respectively, *P* > 0.05, ANOVA, *n* = 6; Fig. 7).

#### LDH activity in media

**Fig. 8:**
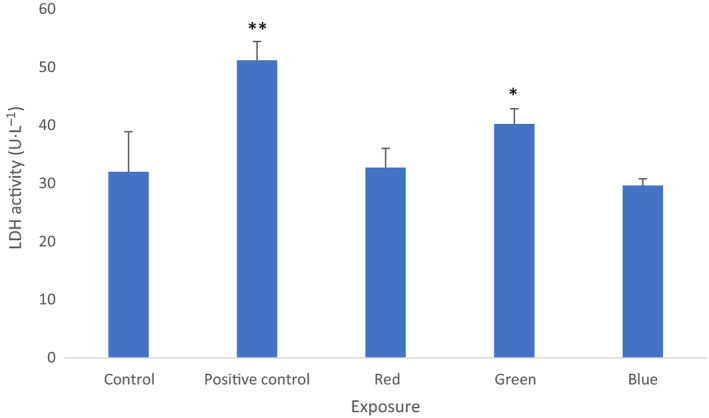
LDH activity in culture media. There is a significant increase following the green range light irradiance of cells in comparison with the controls (*P < 0.05, **P < 0.001, *n* = 6). Mean values with standard deviation (vertical bars) are presented. Filtered light predominant irradiance: red—593–840 nm, green—560–650 nm, blue—420–580 nm, control—cells kept in dark, positive control—cells kept in dark and treated by FGIN‐1‐2710^‐5^ m.

The green range light irradiance caused a significant increase in LDH activity in the media (mean 40.25 ± 2.63 SD U L^−1^ compared to mean 32 ± 6.928 SD U L in the controls, *P* < 0.05, ANOVA, *n* = 6). In samples exposed to the red range light and the blue range light, no difference was found in comparison with the controls (mean 32.75 ± 3.304 SD U/L and 29.667 ± 1.155 SD U/L, respectively, compared to mean 32 ± 6.928 SD U/L in the controls, *P* > 0.05, ANOVA, *n* = 6; Fig. 8).

To simplify the interpretation, the results are summarized in Table 1.

**Table 1:** Summary of cellular response to the investigated LED irradiance. NA, not applicable.

Stage 1	Microscopic cell counting	Cytometric viable cells number	LDH activity in culture media	ALP activity in cells	Osteocalcin content in cells
10 Hz	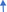	NA	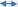	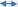	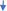
20 Hz	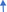	NA	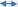	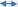	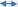
30 Hz	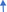	NA	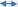	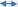	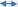
40 Hz	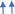	NA	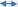	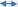	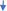
Stage 2					
Red Irradiance	NA	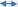	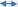	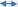	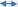
Green Irradiance	NA	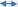	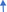	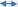	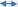
Blue Irradiance	NA	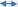	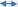	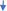	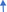

## Discussion

We investigated pulsed LED light effects on human osteoblast‐like cells *in vitro*. The frequency range of 10–40 Hz was chosen according to a previous study showing that osteoblastic‐like cells are metabolically sensitive to mechanical [13] and electromagnetic [14] stimulation at similar frequencies. Our results showed that there was positive cell growth in all cultures tested, but the 40 Hz group had a significantly higher number of cells compared to the controls. It could then be assumed that the higher frequencies of cell irradiance have a more pronounced effect on increasing the number of cells in the exposed cultures.

Thus, our results show that irradiance of cells by 40 Hz frequency by light from an LED source causes the most prominent cellular effects, for example, higher proliferation rate and lower cellular synthetic activity, that is, a significant decrease in cellular osteocalcin content without affecting cellular death rate and maturation level, which were measured by cellular ALP and LDH activity in the media, respectively. These findings might suggest that a minimal threshold frequency of cell irradiance by pulsed white light is required to cause higher proliferation while slowing down cellular synthetic activity, without a prominent effect on cellular maturation. Previous evidence of LED irradiation affecting the mitogenic signaling pathway in mouse fibroblasts *in vitro* [26] via the cytochrome C might suggest a cellular pathway of this phenomenon [2, 27].

We are aware of a previous report that showed an opposite cellular activity when irradiance of MSCs by multiple doses at 15 mW·cm^−2^ and 4 J·cm^−2^ caused a parallel cellular increase in ALP activity and osteocalcin content [28]. Since the experimental setups are not identical (we used up to two order of magnitude lower range of irradiance), a direct comparison of results is difficult, but the general differences between the two are intriguing and might suggest the existence of as yet unknown factors and pathways that might be involved in the visible light range of irradiance transduction into cellular pathways. These should be investigated further in future studies. Several previous reports show increased osteoblast proliferation *in vitro* by LED photobiomodulation at a high irradiance intensity in the blue (wavelength above 630 nm) spectrum at least and above 16 mW/cm^2^ in continuous not pulsed protocols [29, 30, 31, 32]. In the present report, a narrow range of 40 Hz‐pulsed low‐intensity photobiomodulation in the green spectrum (diffuse transmittance 560–650 nm, maximal cell irradiance 0.4 mW/cm^2^) showed a similar stimulation of osteoblast proliferation. This indicates that the green range of irradiance elicits the photobiomodulation of cell proliferation at a much lower irradiance intensity, suggesting the existence of the threshold values of osteoblast photobiomodulation by a low‐intensity‐pulsed irradiance.

Overall, Stage 1 of this study showed that the LED irradiance of cultured human osteoblast‐like cells with a maximal intensity of 2.4–2.5 mW/cm^2^ when applied at a 40 Hz frequency caused a prominent increase in cell proliferation with a parallel inhibition of cellular synthetic activity, suggesting a shift in the cell cycle toward the mitogenesis. From these results, a logical question might arise regarding the existence of specific visual light spectra ranges that might be responsible for enhancing the cell proliferation rate, cell death rate, and cellular synthetic activities. This question is important for clarifying the existence of one or possibly several pathways involved in these cellular responses that we found had evolved following pulsed 40 Hz cellular irradiance. To further verify this uncertainty, in Stage 2 of the experiments, we investigated the influence of pulsed 40 Hz LED light irradiance of cells at different ranges of visible spectrum. As in Stage 1, the human osteoblast‐like cells were evaluated for their number, cellular maturation (ALP activity), cellular synthetic activity (cellular *osteocalcin* content), and cell death rate (LDH activity in culture media). We found an increased proliferation of cells following exposure to the intermediate spectrum of green light (560–650 nm); that is, there was no difference in cell numbers following green light irradiance in comparison with other irradiance conditions, with a parallel significant increase in cell death rate (a significantly higher level of LDH activity in culture media). This phenomenon was not found following exposure to the pulsed LED 40 Hz light at the edges of the visible spectrum, that is, by the red and blue irradiance.

The 40 Hz‐pulsed LED blue range (420–580 nm) light irradiance caused a significantly lower rate of cell maturation (significantly decreased ALP activity) but increased synthetic activity (according to the significantly higher *osteocalcin* content in the cells). This apparent paradox, that is, decreased cellular maturation with a higher synthetic activity of the cells, might indicate different cellular pathways that react independently to the light irradiance. These pathways should be investigated further in future studies.

The results of the Stage 2 experiment show that only the blue range irradiance affected the *osteocalcin* secretion by cells (a significant increase in the content of culture media without a difference in cell numbers in comparison with controls). This finding is not consistent with the Stage 1 results, which showed an opposite effect—an overall significant decrease in *osteocalcin* secretion following 40 Hz irradiance. We can attribute these differences to the cumulative effects of light in different spectra ranges that have a synergistic or threshold, but not additive, effect on the cellular pathways. This assumption is consistent with a previous study [29] showing an increase in *osteocalcin* content in rat mesenchymal cells following irradiance with white light in multiple doses at 16 mW/cm^2^ and 4 J/cm^2^. These irradiance values are two orders of magnitude higher than the irradiance used in the current report, suggesting the existence of threshold values of irradiance that can elicit or shut down cellular synthetic activity. Naturally, these controversial phenomena should be investigated further in future studies.

We are aware that we did not use ‘pure’ spectra; that is, both the green and blue range irradiances contain red to far‐red wavelengths, but because the red range had no significant effect on any of the parameters measured, we can assume that the red impurity had no effect on the results in the green and blue spectrum range of irradiance.

The results of this study show that 40 Hz‐pulsed LED green light (560–650 nm) irradiance causes an increase in human osteoblast‐like cell numbers with a parallel increase in cell death rate. Additionally, a paradoxical effect inconsistent with the observations on similarly applied white LED irradiance of 40 Hz‐pulsed LED blue range light (420–580 nm) irradiance caused a parallel decrease in osteoblast maturation and an increase in osteoblast synthetic activity. Accordingly, due to the paucity of published data on the effects of pulsed visible light on the human normal cell metabolism, the current results should encourage further research in order to verify the mechanisms and cellular pathways that are affected by pulsed visible light irradiance.

This is a study on an *in vitro* cellular level. The foreseen application of osteoblast stimulation by visible light irradiance might be utilized in *in vitro* tissue engendering techniques of bone generation for clinical use as a bone graft [33].

## Conclusions

In this report, we show for the first time that the green range spectrum of pulsed white light irradiance affects cell proliferation and cell death, and the blue range spectrum primarily affects cellular maturation and metabolism. This phenomenon is probably related to the different energy levels of these spectrum irradiances and might indicate differences in energy consumptions of the different cellular pathways. These differences are measurable and can be used as a simple method for measuring energy requirements of different cellular biochemical pathways.

## Conflict of interest

The authors declare no conflict of interest.

## Author contributions

NR designed and executed the experiments, processed results, wrote and proofread the manuscript. GR executed the experiments, processed results, and proofread the manuscript. NN executed the experiments, processed results, and proofread the manuscript.
